# H_2_+CO_2_ Synergistic Plasma Positioning Carboxyl Defects in g-C_3_N_4_ with Engineered Electronic Structure and Active Sites for Efficient Photocatalytic H_2_ Evolution

**DOI:** 10.3390/ijms23137381

**Published:** 2022-07-02

**Authors:** Daqian Wang, Zhihao Zhang, Shuchuan Xu, Ying Guo, Shifei Kang, Xijiang Chang

**Affiliations:** 1 College of Science, Donghua University, Shanghai 201620, China; 2202230@mail.dhu.edu.cn (D.W.); 2212289@mail.dhu.edu.cn (S.X.); guoying@dhu.edu.cn (Y.G.); 2 Department of Environmental Science and Engineering, University of Shanghai for Science and Technology, Shanghai 200093, China; zhangzhihao210@163.com (Z.Z.); sfkang@usst.edu.cn (S.K.); 3 Magnetic Confinement Fusion Research Center of Ministry Education, Donghua University, Shanghai 201620, China

**Keywords:** g-C_3_N_4_, carboxyl group, defect engineering, non-thermal plasma method, CO_2_ utilization

## Abstract

Defective functional-group-endowed polymer semiconductors, which have unique photoelectric properties and rapid carrier separation properties, are an emerging type of high-performance photocatalyst for various energy and environmental applications. However, traditional oxidation etching chemical methods struggle to introduce defects or produce special functional group structures gently and controllably, which limits the implementation and application of the defective functional group modification strategy. Here, with the surface carboxyl modification of graphitic carbon nitride (g-C_3_N_4_) photocatalyst as an example, we show for the first time the feasibility and precise modification potential of the non-thermal plasma method. In this method, the microwave plasma technique is employed to generate highly active plasma in a combined H_2_+CO_2_ gas environment. The plasma treatment allows for scalable production of high-quality defective carboxyl group-endowed g-C_3_N_4_ nanosheets with mesopores. The rapid H_2_+CO_2_ plasma immersion treatment can precisely tune the electronic and band structures of g-C_3_N_4_ nanosheets within 10 min. This conjoint approach also promotes charge-carrier separation and accelerates the photocatalyst-catalyzed H_2_ evolution rate from 1.68 mmol h^−1^g^−1^ (raw g-C_3_N_4_) to 8.53 mmol h^−1^g^−1^ (H_2_+CO_2_-pCN) under Xenon lamp irradiation. The apparent quantum yield (AQY) of the H_2_+CO_2_-pCN with the presence of 5 wt.% Pt cocatalyst is 4.14% at 450 nm. Combined with density functional theory calculations, we illustrate that the synergistic N vacancy generation and carboxyl species grafting modifies raw g-C_3_N_4_ materials by introducing ideal defective carboxyl groups into the framework of heptazine ring g-C_3_N_4_, leading to significantly optimized electronic structure and active sites for efficient photocatalytic H_2_ evolution. The 5.08-times enhancement in the photocatalytic H_2_ evolution over the as-developed catalysts reveal the potential and maneuverability of the non-thermal plasma method in positioning carboxyl defects and mesoporous morphology. This work presents new understanding about the defect engineering mechanism in g-C_3_N_4_ semiconductors, and thus paves the way for rational design of effective polymeric photocatalysts through advanced defective functional group engineering techniques evolving CO_2_ as the industrial carrier gas.

## 1. Introduction

Preparation of high-performance photocatalytic materials by using non-metallic elements (such as C, N, O) with rich reserves and no secondary pollution is an ideal way to realize solar energy clean conversion [1,2]. Graphitic carbon nitride (g-C_3_N_4_), a unique 2D layered non-metallic material, has an energy band structure that is very suitable for the two key semi-reaction steps of photocatalytic water splitting and hydrogen production. It also has high thermal stability and can be synthesized easily. Therefore, it is widely regarded as a photocatalytic material with broad application prospects in photocatalytic decomposition of aquatic hydrogen and artificial photosynthesis. It has important research value in the fields of organic pollutant degradation and carbon dioxide reduction. However, at present, g-C_3_N_4_ still faces problems such as the serious recombination of photo-generated charge carriers, unfavorable electronic structure and limited active sites [3,4]. How to regulate its melon structure and electronic structure for better energy and environmental applications has become one of the research hotspots in this field. 

Recent research shows that introducing defects and functional groups into the triazine structural unit of g-C_3_N_4_ is one effective way to solve the above problems. However, the reported methods of introducing nitrogen defects usually involve multi-step operation and harsh reaction conditions (such as high-temperature treatment in the reducing atmosphere). The experimental process is dangerous and unapplicable at a large scale. More importantly, most of the defects are uneven surface defects, and the degree of defects uncontrollable, which is very unfavorable for accurately controlling the electronic structure of g-C_3_N_4_. Therefore, more refined and integrated defect control strategies are needed.

Surface functionalization can improve the photocatalytic performance of g-C_3_N_4_ by adjusting and optimizing its basic structural units (molecular level), which is significantly different from the strategy of heterojunction construction [5,6]. Surface functionalization mainly involves functional group modification and surface defect modification. In the former, adjusting the molecular structure of g-C_3_N_4_ to expand its light response and reduce photoinduced charge recombination is an effective method to improve the photocatalytic performance of g-C_3_N_4_. Given the organic properties of the g-C_3_N_4_ conjugated structure, g-C_3_N_4_ photocatalysts can be very feasibly prepared by adjusting the molecular composition through copolymerization [7,8]. Noticeably, the diversity of organic reactions provides various methods to design supramolecules for modifying g-C_3_N_4_ with nitrogen-rich precursors and comonomers. The surface properties, texture and electronic structure of g-C_3_N_4_ can be optimized by introducing special functional groups into the g-C_3_N_4_ conjugate system. Meanwhile, surface defect modification of g-C_3_N_4_ can effectively enhance charge separation, optimize energy band structure and broaden light response range. Therefore, in recent years, various surface defects, such as carbon vacancy, nitrogen vacancy, cyanamide defect and structural edge defect, have been widely studied to improve the photocatalytic performance of g-C_3_N_4_. In general, it is better to integrate the advantages of the above two defect types, such as ring opening defect carboxyl structure. However, because conventional chemical methods easily cause excessive oxidation corrosion, it is very challenging to prepare ring opening defective or carboxyl endowed carbon nitride using a green and industrialized process [9,10]. 

One important and reasonable idea of preparing carbon nitride photocatalysts modified by defective functional groups is to first introduce point defects and then graft functional groups on point defects. Clearly, the introduction of point defects, whether holes or dopants, is powerful in modifying the surface, optical and electrical properties of g-C_3_N_4_, and thus improves the performance of photocatalysis in water decomposition, carbon dioxide reduction and nitrogen fixation [11]. Generally, g-C_3_N_4_ nanomaterials will have some significant changes after the introduction of point defects, such as (1) tunable band gap, (2) defect-induced intermediate gap, (3) larger surface area, (4) inhibited recombination of photogenerated electrons and holes, and (5) improved adsorption and activation of reactant molecules. More importantly, our previous work shows that functional group modification is unstable in most cases if the functional groups are directly grafted. For example, the carboxyl group has a large space station [12]. If there is no point vacancy configuration, high-quality defect functional group modification can be hardly achieved.

At present, there is a lack of reliable schemes and techniques for accurate and unified engineering designing of defects. Noticeably, the common synthesis problem of g-C_3_N_4_ is its proneness to aggregation, which requires a layered process to achieve the uniform introduction of defects. In addition, the developed synthesis method must be low-cost and scalable to allow the scaled-up production of controllable defective catalysts, which is still a great challenge to date. At the same time, the relationship between defects and the photocatalytic activity of different forms of g-C_3_N_4_ needs more exploration, which is worth research from experimental, calculational and analytical perspectives.

Herein, we designed a low-temperature plasma technology that mainly used CO_2_ resource to achieve defect site regulation and carboxyl structure introduction in carbon nitride in one step. Previous research on CO_2_/H_2_ plasma confirms that the mixing of CO_2_ and H_2_ directly influences the plasma parameters and results in a large fraction of H atoms and carboxyl precursor species [13,14]. Both optical emission spectroscopy and quadrupole mass spectrometry can detect H species (through the lines Hα, Hβ, and Hγ), CO, CO_2_, CO_2_^+^, O_2_, OH, O, C_2_, CO, and CO^+^ [15,16]. These plasma components provide many possibilities for optimizing the surface properties and electronic structure of polymer semiconductors towards rational carboxyl-defective modification, but have not been studied in the rational design and synthesis of functional group defect-endowed g-C_3_N_4_. A highly ionized H_2_+CO_2_ plasma environment was developed using a rational microwave surface wave plasma approach and employed for rational carboxyl defect regulation of g-C_3_N_4_. In this study, the changes in sample morphology, functional group structure and photoelectric properties after plasma treatment were analyzed by various characterizations. Together with density functional theory (DFT) calculation, the mechanisms of electronic structure optimization and surface-active site improvement were analyzed. In addition, the necessity for simultaneous use of hydrogen and carbon dioxide plasma and the potential of non-thermal low-temperature plasma technology in dealing with polymer semiconductor defect structures were discussed.

## 2. Results and Discussion

### 2.1. DFT Calculation of Carboxyl-Defective g-C_3_N_4_

Nowadays, g-C_3_N_4_ polymeric semiconductors show intriguing prospects by virtue of rich sources, high stability, and easy regulation. Nevertheless, an ambiguous understanding about the structural and electronic modulation processes of this fascinating material will inevitably hamper further progress. Therefore, the DFT calculation of ideal carboxyl-defective g-C_3_N_4_ was performed to better regulate molecular structure design. Previous studies on carboxyl defect materials mostly focus on carbon materials similar to carbon nitride materials. The introduction of oxygen-containing functional groups such as carboxyl groups into carbon materials can regulate the catalytic activity by changing the local properties of the catalyst. However, the roles of different oxygen-containing functional groups in catalytic ozonation and quantitative structure–activity relationships of carbon materials are still unclear. Moreover, previous theoretical calculations ignored the possibility and stability of g-C_3_N_4_ configuration.

**Figure 1:**
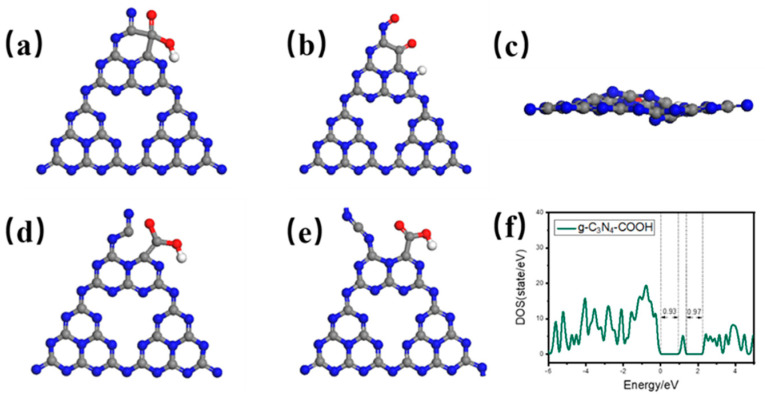
The DFT modeling closed-loop and open-loop structure of carboxyl-defective g-C_3_N_4_ at N vacancy point defect site (gray, blue, pink and red spheres: C, N, Cu and O, respectively). (**a**) Original closed-loop configuration and (**b**) unsubstantiated structure after evolution with (**c**) 2D in-plane top view structure; (**d**) original open-loop configuration and (**e**) rational structure after evolution with (**f**) corresponding total density of states.

Based on the common sense that hydrogen plasma easily produces N-site defects [17,18], we determined a variety of possible carboxyl defect structures of g-C_3_N_4_ through DFT calculation (Figure 1). In the typical closed-loop state, the evolutionary structure is unstable after simulation, which is manifested in the disintegration of the carboxyl structure, and the two-dimensional plane structure cannot be maintained. Therefore, we believe that the open-loop defect structure of g-C_3_N_4_ is favorable [19]. Many evolutionary simulations demonstrate that the structure designed in Figure 1d,e is relatively stable, and the open-loop state finally evolves into a local stable state with two-dimensional plane stability. Calculation of the density of states of the optimized structure shows that despite no large change in the main energy band structure, there is an obvious intermediate energy level around 1.15 eV, which is mainly caused by the change of N 2S orbit. Therefore, we believe that plasma treatment is suitable for producing carboxyl defect sites with open ring structure. From the perspective of DFT, this defect site is conducive to optimizing the electronic structure of the material and is expected to provide more surface-active sites, especially for the energy level requirements of photocatalytic hydrogen production. 

Introducing nitrogen deficiency into the framework of g-C_3_N_4_ as the critical prepositioned point defects for the subsequent carboxyl grafting is one effective solution to the catalyst preparation problems, so it has attracted extensive attention from researchers. However, the reported methods of introducing nitrogen defects usually involve a multi-step operation, and need harsh reaction conditions (such as reducing atmosphere or high temperature treatment), which are dangerous in the experimental process, practically inapplicable and cannot control the degree of defects. Therefore, how to prepare g-C_3_N_4_ with controllable nitrogen defect by a simple synthetic method and thus to further improve the photocatalytic activity is of great research significance [20,21]. Meanwhile, effective and mild treatment of carboxyl defects without affecting the main structure of materials is also a major challenge in the regulation of carboxyl defects. As an electroless discharge, the microwave surface wave plasma technique described above can form large-area uniform high-quality joint H_2_+CO_2_ plasma, which has the potential of large-scale industrial application. Furthermore, the microwave surface wave discharge mode used here is expected to show great potential in rational regulation of carboxyl defects of polymer semiconductor photocatalysts and in sustainable CO_2_ utilization, in addition to the traditional dielectric-barrier discharge mode. CO_2_, a cheap, non-flammable and non-explosive gas, is a practical choice from economic and safety perspectives. Moreover, since CO_2_ is considered to be the primary greenhouse gas contributing to global warming, the conversion of CO_2_ molecules into high-value-added products is of great importance for fundamental research and industrial applications.

**Figure 2:**
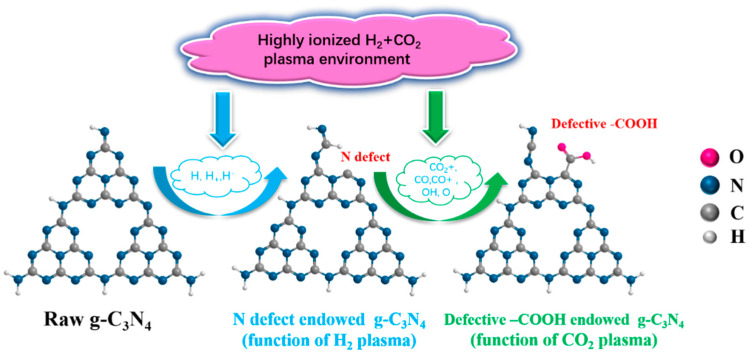
The proposed schematic diagram of preparing carboxyl-defective g-C_3_N_4_ by introducing prepositioned point defects through joint H_2_+CO_2_ plasma.

Figure 2 illustrates the proposed schematic diagram of preparing carboxyl-defective g-C_3_N_4_ by introducing prepositioned point defects through joint H_2_+CO_2_ plasma. The core concept to endow defective functional groups is to first introduce point defects by H_2_ plasma and then in situ graft functional groups on point defects by CO_2_ plasma, which have been verified to be useful in the modulation of defective carbon nitride materials for electrochemical applications [22]. 

### 2.2. Morphology

**Figure 3:**
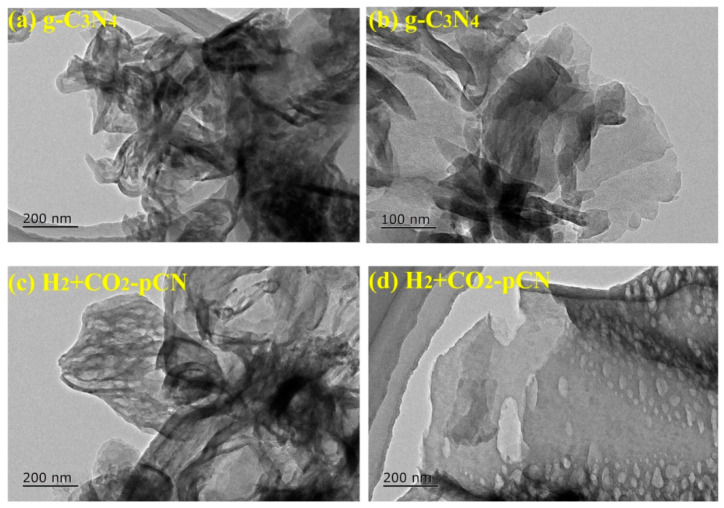
TEM images of raw g-C_3_N_4_ and H_2_+CO_2_ plasma treated g-C_3_N_4_ (H_2_+CO_2_-pCN).

On the basis of theoretical calculations and simulations, we used the modulated microwave nonthermal plasma equipment to gently and quickly modify the materials. Figure 3 displays the typical TEM images of raw g-C_3_N_4_ and H_2_+CO_2_ plasma-treated g-C_3_N_4_ (H_2_+CO_2_-pCN). The raw g-C_3_N_4_ has an irregularly sized and thick-layer-stacked structure (Figure 3a,b). Interestingly, the TEM images of H_2_+CO_2_-pCN (Figure 3c,d) show a stretched graphene-like nanosheet structure with many mesopores. The morphological changes of g-C_3_N_4_ photocatalysts before and after the H_2_+CO_2_ plasma treatment are due to the shear effect of mixed plasma species of H (through the lines Hα, Hβ, and Hγ), CO, CO_2_, CO_2_^+^, O_2_, OH, O, C_2_, CO, and CO^+^. The multilayer nanobelt structure of H_2_+CO_2_-pCN reveals a large number of active edge sites, which can realize multiple visible-light reflection and scattering to improve energy utilization efficiency, and have a large interlayer distance, which is conducive to mass transfer [23,24].

### 2.3. Physicochemical Characterization

**Figure 4:**
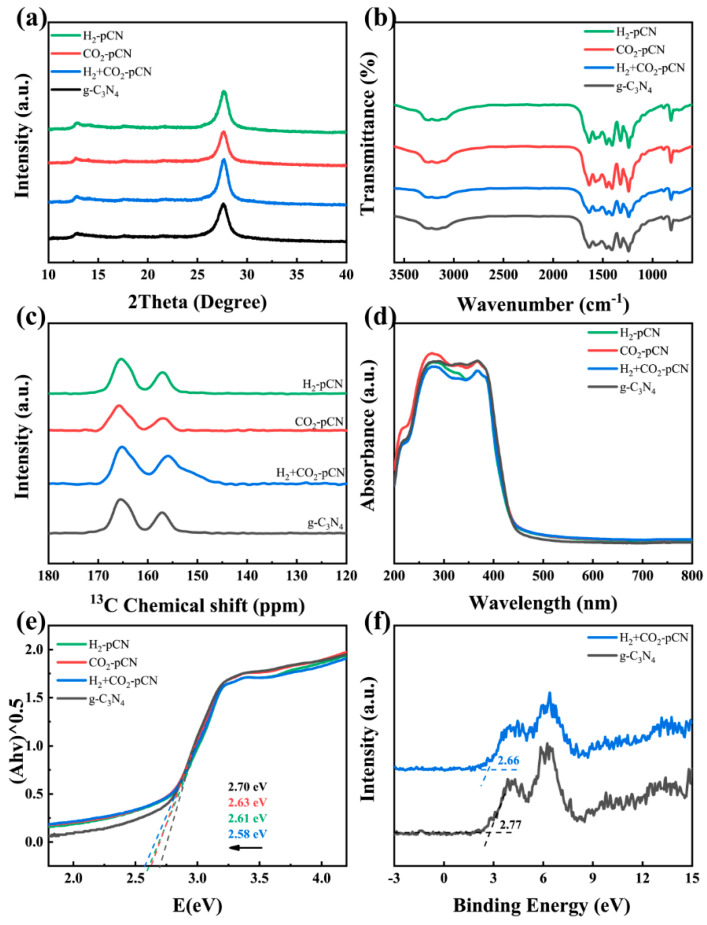
(**a**) XRD patterns, (**b**) FTIR spectra, (**c**) solid-state 13C MAS NMR spectra, (**d**) UV-vis DRS spectra and (**e**) transformed Kubelka–Munk function versus proton energy plots of raw g-C_3_N_4_, H_2_-pCN, CO_2_-pCN, and H_2_+CO_2_-pCN. (**f**) XPS valence band spectra of raw g-C_3_N_4_ and H_2_+CO_2_-pCN.

All the raw g-C_3_N_4_ samples were synthesized via urea thermal polymerization. The crystal structure of the polymer semiconductor g-C_3_N_4_ was investigated by XRD patterns (Figure 4a). Specifically, the two main peaks around 13.1° and 27.7° can be indexed to (100) interplanar structural packing and (002) interlayer stacking peaks, respectively. Different types of plasma treatments basically did not change the polymerization structure of the material, indicating that the method has high mildness and will not excessively oxidize or corrode the material or destroy its photoelectric properties and surface-active sites. FTIR was further used to identify the potential surface functional group structure. The multiple absorption peaks at 1200–1650 cm^−1^ in Figure 4b correspond to the stretching vibrations of N=C and N–(C)_3_ in the CN heterocyclic ring, and the sharp peak at 814 cm^−1^ is attributed to the respiratory vibration of the triazine structural unit [25]. The triazine structure of the g-C_3_N_4_ matrix was maintained after the plasma treatment. The multiple broad peaks at 3000–3500 cm^−1^ belonging to N-H, –OH and –COOH stretching vibrations slightly changed in the plasma-treated sample, which may be evidence for the reformation of carboxyl-related structures. The most important evidence for the successful introduction of carboxyl groups comes from solid-state 13C NMR (Figure 4c). All samples contain two strong peaks at 165.8 and 156.3 ppm (Figure 4c), which correspond to the characteristic C_3N_ and C_2N–NHx_ atoms in the heptazine units of g-C_3_N_4_, respectively. In addition, a new broad peak at about 147–155 ppm emerges only in H_2_+CO_2_-pCN, which belongs to the C atoms in –COOH. These structural and functional group characterization results indicate that the H_2_+CO_2_ plasma method is useful in developing carboxyl-defective g-C_3_N_4_ photocatalysts [26,27]. Noticeably, none of the H_2_-pCN and CO_2_-pCN control samples show carboxyl properties, which indicates the necessity of the joint H_2_+CO_2_ plasma mode.

**Figure 5:**
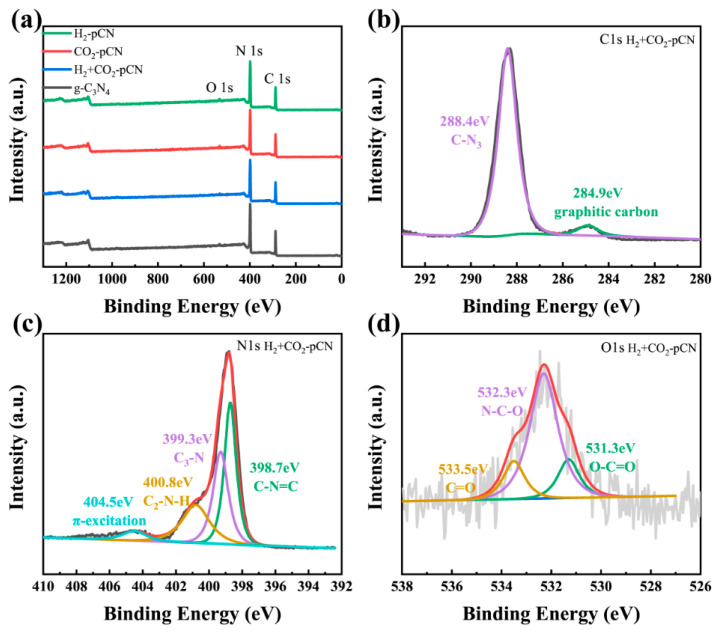
(**a**) XPS survey spectra of raw g-C_3_N_4_, H_2_-pCN, CO_2_-pCN, and H_2_+CO_2_-pCN. (**b**) C 1s XPS, (**c**) N 1s XPS, and (**d**) O 1s XPS spectra of the optimal H_2_+CO_2_-pCN.

The UV-vis spectrum exhibits an optical absorption edge around 430 nm (Figure 4d). On the basis of the above structural characterizations, we conclude that a typical polymeric carbon nitride with a tri-s-triazine-based structure is successfully obtained. The optical properties and light-harvesting abilities of g-C_3_N_4_ samples were slightly modified by the unique H_2_+CO_2_ plasma treatment. The bandgaps and band structure of g-C_3_N_4_ were illustrated according to the electronic bandgaps determined from the transformed KubelkaMunk function in Figure 5e as well as the XPS valence band spectra of raw g-C_3_N_4_ and H_2_+CO_2_-pCN (Figure 4f). The narrowed bandgap of H_2_+CO_2_-pCN (2.58 eV) compared with the initial state of raw g-C_3_N_4_ (2.70 eV) reveals the enhanced visible-light harvesting ability and electronic structure of the carboxyl deficient g-C_3_N_4_ [28].

XPS demonstrates that the chemical compositions of g-C_3_N_4_ both before and after plasma treatment are mainly carbon and nitride as well as a tiny signal of O 1s, which was slightly enhanced after the H_2_+CO_2_ plasma treatment (Figure 4f). The calculated surface O/C atomic ratio increases from 0.021 to 0.043 during the plasma process, which aligns with the introduction of carboxyl groups verified by 13C NMR. High-resolution C 1s (Figure 5b), N 1s (Figure 5c) and O 1s (Figure 5d) spectra of H_2_+CO_2_-pCN were investigated to further reveal the defect structure and chemical composition of H_2_+CO_2_ plasma-treated g-C_3_N_4_. The C 1s spectrum exhibits two main peaks centered at 284.8 and 288.4 eV, which are assigned to the graphitic carbon and the sp^2^-bonded carbon of the tri-s-triazine-based structure, respectively. The N 1s spectrum shows four binding peaks at 398.7, 399.3, 400.8 and 404.5 eV, representing bi-coordinated N (C–N=C), tri-coordinated N (N-3C), amino N (C–NHx, x = 1,2) and π-excitation in the framework, respectively [29]. The strong amino N in H_2_+CO_2_-pCN indicating more exposed edges was obtained for fast charge separation. The O 1s XPS spectra of H_2_+CO_2_-pCN show clear carboxyl properties by an enriched O–C=O signal at 531.3 eV, which is invisible in raw g-C_3_N_4_ samples [30]. All the XPS results show that the carboxyl defects are preferentially formed, and the mechanism may be related to the deficient N point site, which is consistent with the theoretical calculation and experimental design. In our previous work using ammonia plasma and oxygen plasma, such an obvious beneficial carboxyl structure has never been observed, so this result is exciting.

### 2.4. Electronic and Electrochemical Properties

**Figure 6:**
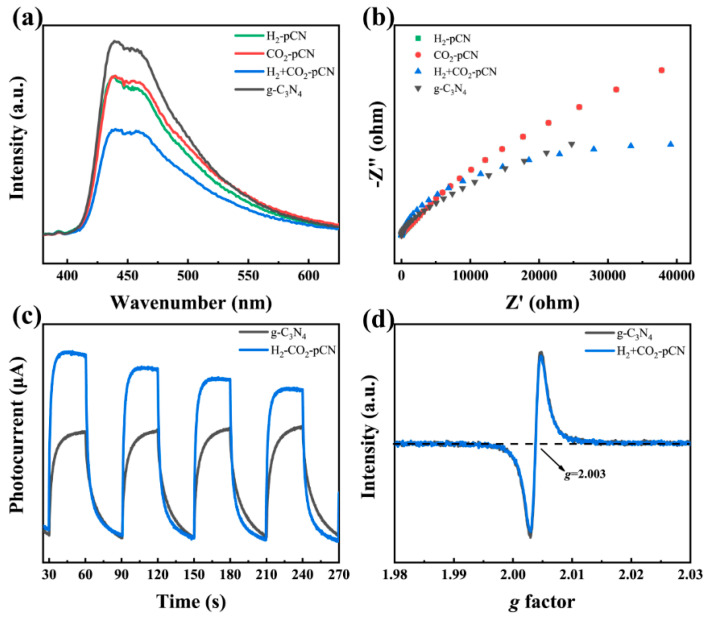
(**a**) PL and (**b**) EIS spectra of all samples. (**c**) Photocurrent curves and (**d**) EPR spectra of raw g-C_3_N_4_ and H_2_+CO_2_-pCN.

MCNN has higher photogenerated electron hole separation efficiency than g-C_3_N_4_ (Figure 6a,c), which can be attributed to the electron repositioning caused by the carboxyl defects. After the H_2_+CO_2_ plasma treatment, the fluorescence signal decreases gradually, indicating the introduction of carboxyl group is conducive to the rapid radiation electron hole recombination at the band tail of carboxyl defects. The electrochemical impedance diagram shows that the charge transfer of carboxyl deficient g-C_3_N_4_ is enhanced in comparison with the original g-C_3_N_4_. The valid evidence of more obvious carrier separation comes from the significantly enhanced photocurrents of H_2_+CO_2_-pCN over the raw g-C_3_N_4_ (Figure 6c) [31,32]. EPR was employed to further study the state of delocalized electrons on the π–conjugated aromatic ring of g-C_3_N_4_, which showed the same g value. This result suggests the electronic structure after H_2_+CO_2_ plasma relocation is still highly covalent rather than delocalized, indicating that plasma treatment is a conservative carboxyl structure regulation, which is different from other reported chemical methods.

### 2.5. Visible-Light-Driven H_2_ Evolution Performances and Optimization Mechanism

**Figure 7:**
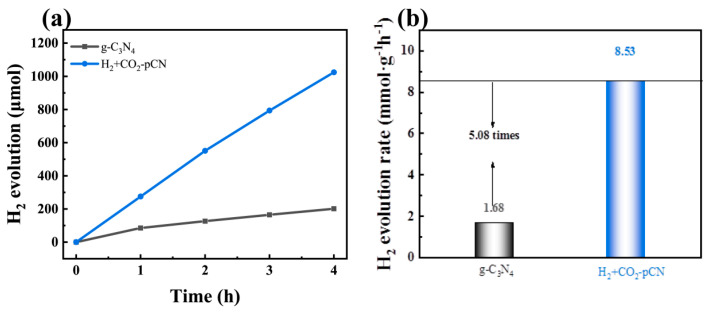
(**a**) Time course of H_2_ evolution and (**b**) rates of the photocatalytic H_2_ evolution over 5 wt.% Pt-loaded g-C_3_N_4_ before and after H_2_+CO_2_ plasma treatment in 10 vol% TEOA solution under visible-light irradiation (λ ≥ 380 nm) (30 mg of the catalyst used in each experiment).

The above physiochemical and photoelectric performance tests confirm that only the H_2_+CO_2_ plasma-treated g-C_3_N_4_ samples (H_2_+CO_2_-pCN) are endowed with the proposed carboxyl structure. A simple photocatalytic hydrogen production experiment was carried out (Figure 7a,b) to further verify the properties of electronic structure and active site regulation. The gas chromatography determined H_2_ evolution rates over the 5 wt% Pt loaded g-C_3_N_4_ samples before and after H_2_+CO_2_ plasma treatment were 1.68 and 8.53 mmol h^−1^g^−1^, respectively. The photocatalytic H_2_ evolution results reveal the significant photocatalytic performance enhancement of H_2_+CO_2_ plasma-treated g-C_3_N_4_, which was empowered by the rational CO_2_ plasma generated carboxyl defective structure based on the H_2_ plasma generated nitrogen point-deficient g-C_3_N_4_. The H_2_+CO_2_-pCN produced by this facile in situ plasma synthesis strategy also shows a high apparent quantum yield (AQY) of 4.14% at 450 nm, which is close to industrial utility [33]. 

**Figure 8:**
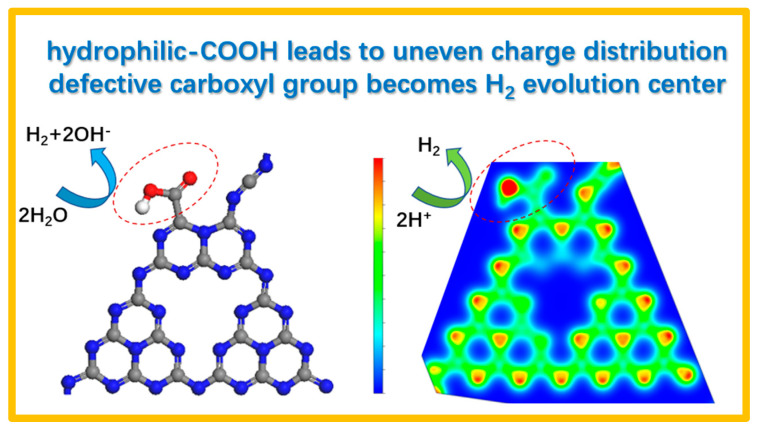
Schematic diagram of the dual-enhancement mechanism of structural carboxyl defect sites on the promoted H_2_ production activity of g-C_3_N_4_. Left: structure diagrams of defective carboxyl site with hydrophilic properties facilitating the H_2_O to H_2_ reaction; right: corresponding charge distribution map with electron-rich site facilitating the H^+^ to H_2_ reaction.

With the help of theoretical calculation, we believe the improvement of hydrogen production performance of photocatalysts prepared by the plasma method is due to the favorable hydrophilic sites [34,35] and rearranged electronic structure [36,37] caused by carboxyl defects (Figure 8). The carboxyl defect sites may also promote the binding state of Pt cocatalysts for H_2_ generation. As a result, more active sites for hydrogen production were developed, and thus both the H_2_O to H_2_ reaction and the H^+^ to H_2_ reaction were boosted, leading to the superior H_2_ evolution performance. 

Based on the above experimental results, we believe that the plasma modification strategy proposed in this paper can effectively control the defect structure and electronic structure of g-C_3_N_4_ material to promote its photocatalytic activity. Although the introduction of appropriate carboxyl groups is beneficial to the g-C_3_N_4_ polymer semiconductor, the preparation methods are limited long-term [10,38]. The non-thermal microwave plasma method developed here is able to process samples at the kilogram level within minutes in the laboratory stage [39,40], and thus is promising in the rational design and low-cost, scaled-up preparation of structure precisely modulated materials for industrial application. 

## 3. Materials and Methods

### 3.1. Materials

Analytical grade urea obtained from Aladdin Industrial Corp. (Shanghai, China) was used as the precursor of g-C_3_N_4_ and used without further purification. High-purity hydrogen gas (containing 95% Ar as safety carrier gas) and high-purity carbon dioxide gas were purchased from Qingkuan Corp. (Shanghai, China).

### 3.2. Preparation of Raw g-C_3_N_4_ and Carboxyl-Defective g-C_3_N_4_

Raw g-C_3_N_4_ was synthesized by direct thermal polycondensation of 10.0 g of urea, which was put in a covered crucible and placed in muffle furnace at 560 °C for 4 h at a heating rate of 5 °C·min^−1^. The resulting light-yellow powder after natural cooling to room temperature was ground to a fine powder in an agate mortar and marked as g-C_3_N_4_. Plasma-treated modified g-C_3_N_4_ samples were all synthesized by plasma immersion in a sample chamber for 3 min. The plasma-treated g-C_3_N_4_ samples were named according to the used plasma source (H_2_-pCN, CO_2_-pCN, H_2_+CO_2_-pCN).

### 3.3. Plasma Equipment and Process

The plasma technique is well recognized to be able to generate very highly ionized reactive plasma species (e.g., electrons, ions, excited atoms), and to enable reactions that cannot be achieved by conventional methods. For decades, CO_2_ plasma has been used as a soft oxidation approach to generate the surface carboxyl group of carbon materials to achieve hydrophilicity or improved electrochemical activity. Besides, dielectric barrier discharge plasma was used to prepare N/C vacancy-embedded g-C_3_N_4_ catalysts in situ under H_2_ atmosphere. These generated plasma species further interact with the polymer surface through possible reactions such as etching, cross-linking, and chemical modification. As a consequence, functional groups with desired surface properties are generated on the surface of polymers, such as –OH, –CO, –NH and –COOH. To date, many plasma processes have been developed. Among them, CO_2_ plasma is capable of adhesion promotion as it can form –COOH on polymer surface, and is somewhat less destructive to polymer backbone chains than conventional oxygen-based plasma. To achieve large-area uniform plasma surface modification in industry, the demand for plasma source is increasing. Due to high dissociation, large area and uniformity, electrodeless microwave plasma is promising in industry, although the equipment is expensive. 

**Figure 9:**
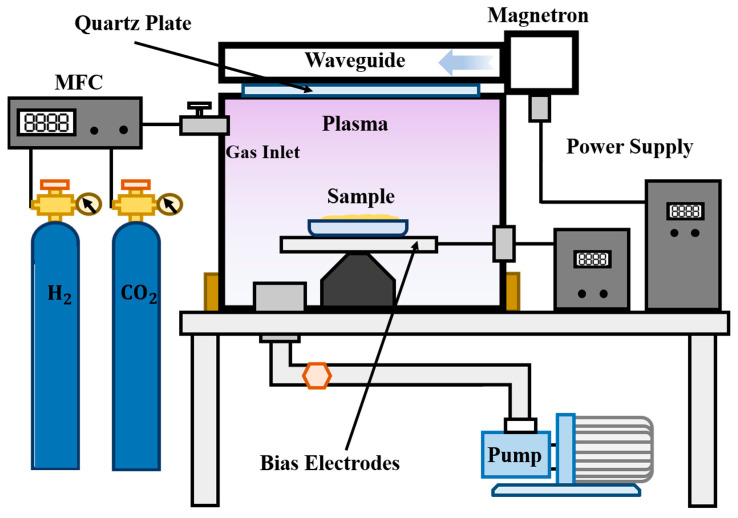
Setup of the microwave plasma equipment capable of joint H_2_+CO_2_ plasma treatment.

For this purpose, we developed a microwave surface wave plasma device shown in Figure 9 to achieve uniform plasma surface modification. The 2.45 GHz microwave was employed as a source to generate surface wave plasma under a quartz window via slot antenna cut on the bottom of waveguide [41]. The working gas system was determined by a vacuum system, cut-off valves, a gas mass flow controller and a gas tank together to create a necessary environment in the processing chamber. When microwave energy was applied, uniform plasma was formed within 1 s. On the sample state, we set an electrode to apply bias voltage to form an accelerating field, which can be used to control the ion energy bombing to the sample. 

### 3.4. Characterization of Carboxyl-Defective g-C_3_N_4_

Morphology of g-C_3_N_4_ photocatalysts before and after the H_2_+CO_2_ plasma modification was recorded by a JEM-2200F transmission electron microscope (TEM) at an accelerating voltage of 300 kV. The polymerization structure of the photocatalysts was determined by an X-ray diffractometer (XRD, Smartlab9K Advance). Fourier transform infrared spectra (FTIR) were recorded with a Nicolet IS5 spectrometer. Solid-state 13C nuclear magnetic resonance (NMR) spectra were acquired on a Bruker Avance III 400 NMR spectrometer. A Thermo Scientific Escalab 250Xi X-ray photoelectron spectroscope (XPS) was run under Al Kα monochromatization to perform XPS elemental analysis and valence spectrum analysis. All ultraviolet-visible (UV-vis) absorption spectra were conducted with a UV-vis absorption spectrophotometer (UV-3600 plus). The photo-electron and hole recombination rates of the photocatalysts were determined by a fluorescence spectrometer (PL, Hitachi FLS1000) at room temperature. Electron paramagnetic resonance (EPR) signals were investigated on a Bruker model EPR A300 spectrometer. Electrochemical impedance spectroscopy (EIS) and transient photocurrents were recorded by a Chi660e electrochemical workstation based on a conventional three-electrode system from frequency 0.01 Hz to 100 kHz at the circuit potential.

### 3.5. DFT Calculation

Spin-polarized DFT calculations were performed using the package CASTEP. The core electrons were treated with ultrasoft pseudopotentials. Given the calculation cost, geometrical optimization was conducted only at the gamma point. After the optimization, the total density of states and differential charge density of the carboxyl defect endowed g-C_3_N_4_ systems were calculated with a cutoff energy of 340 eV and a self-consistent field tolerance of 1 × 10^−6^ eV per atom.

### 3.6. Photocatalytic Degradation Experiment

Visible-light-driven photocatalytic H_2_ production was tested through a 25 °C thermostatic Labsolar-6A system (Perfect Light Company, Beijing, China) with a 300 W Xenon-arc lamp with a 380–420 nm cutoff filter as the light source. A TEOA aqueous solution (10 vol%) was used to provide a sacrificial agent. A H_2_PtCl_6_ solution was used to prepare 5 wt.% Pt-loaded g-C_3_N_4_ samples. After illumination per hour under magnetic stirring, the produced gas was quantified by a Shimadzu GC-2018 gas chromatograph. For the photocurrent measurements, the same Xenon lamp and filter were used, which is consistent with the light source used in the photocatalysis experiments.

## 4. Conclusions

Using semiconductors to absorb solar energy to produce hydrogen from water decomposition is a very effective way to convert solar energy into chemical energy. g-C_3_N_4_ has attracted extensive attention because of its high physicochemical stability, adjustable electronic structure and molecular adjustability. The key to improving the energy application of g-C_3_N_4_ materials is to accurately regulate the structures, so as to modulate photoelectric properties and surface-active sites. At present, most studies focus on enlarging the surface area of catalysts, elemental doping and forming complexes with other (semi)conductors, so as to optimize solar energy utilization. A few attempts have been made to enhance its inherent low activity based on precise molecular tunability strategies. In this work, a unique fast H_2_+CO_2_ plasma immersion treatment approach was developed to enhance the migration and separation of charge carriers. We find the proposed synergistic N vacancy generation and subsequent carboxyl species grafting pathway is reasonable and necessary. The ring opening carboxyl defect structure is considered to be stable and is optimized by DFT calculation, resulting in significant changes in the intermediate energy level. The existence of carboxyl defects in the polymer g-C_3_N_4_ matrix promotes the formation of a porous structure, and exposes more active sites for photocatalytic hydrogen production. In conclusion, the rational H_2_+CO_2_ plasma-treated H_2_+CO_2_-pCN with an optimized electronic structure and active sites shows 5.08-times higher photocatalytic hydrogen production performance. Compared with the ammonia plasmon and oxygen plasma treatment method previously reported by our group, the current work is more in-depth and accurate, and is designed beyond the conventional chemical etching, element doping and functional group regulation. In terms of synthetic method, an in-depth multi-step collaborative structure design is proposed and verified, and systematic DFT calculations and electronic mechanism optimization mechanisms are involved. This study provides fresh understanding about precise molecular regulation of g-C_3_N_4_ via a sustainable and scalable CO_2_ plasma technique.

## Data Availability

The data presented in this study are available on request from the corresponding author.
